# Biological Characterization of 3D-Printed, Sintered Hydroxyapatite Scaffolds Obtained by Fused Filament Fabrication: An In Vitro Study

**DOI:** 10.3390/jfb16100392

**Published:** 2025-10-19

**Authors:** Eddy Shan, Cristina Chamorro, Ana Ferrández-Montero, Rosa M. Martin-Rodriguez, Leire Virto, María José Marín, Begoña Ferrari, Antonio Javier Sanchez-Herencia, Elena Figuero, Mariano Sanz

**Affiliations:** 1 Etiology and Therapy of Periodontal and Peri-Implant Diseases (ETEP) Research Group, Complutense University of Madrid, 28040 Madrid, Spainmarsan@ucm.es (M.S.); 2 Doctorate Programme in Dental Sciences, Complutense University of Madrid, 28040 Madrid, Spain; rm.martin@icv.csic.es; 3 Institute of Ceramics and Glass (ICV), Spanish National Research Council (CSIC), 28049 Madrid, Spainajsanchez@icv.csic.es (A.J.S.-H.)

**Keywords:** in vitro, sintering, tissue engineering, 3D-printed, fused filament fabrication, scaffold, hydroxyapatite, bone regeneration

## Abstract

This study characterized the biological response of MG-63 cells to synthetic, hydroxyapatite scaffolds (HAsint) fabricated via fused filament fabrication. Scaffolds were compared to 2D plate-adherent cultures using six assays: cell morphology and distribution with scanning electron microscopy and confocal laser scanning microscopy; cell proliferation and cytotoxicity via WST-1 tetrazolium assay; relative osteogenic gene expression through reverse-transcription–quantitative polymerase chain reaction, and protein synthesis via multiplex immunoassay. Data were analyzed using one-way ANOVA. Results confirmed high cell viability and uniform distribution on HAsint scaffolds. Proliferation increased significantly over 7 days, though direct cytotoxicity also increased, likely due to the static conditions of the experiment and, subsequently, the high ion reprecipitation from scaffold degradation. Importantly, HAsint scaffolds significantly enhanced osteogenic marker expression of phosphatase alkaline (ALPL), osteopontin (OPN), and osteocalcin (OCN) genes, and elevated concentrations of interleukins (IL)-6, IL-8 and matrix metalloproteinase 1 compared to plate-adherent controls. It can be concluded that 3D-printed HAsint scaffolds support robust osteogenic differentiation and proliferation despite inducing a transient cytotoxic response in vitro. The marked upregulation of key osteogenic genes and proteins confirms the scaffolds’ bioactivity and highlights their potential for bone tissue engineering applications.

## 1. Introduction

The persistent limitations of conventional bone grafts, related to insufficient osteoinductivity and angiogenic capacity, biological safety, donor morbidity, volumetric instability, clinical availability, and cost-effective manufacturing, have driven the search for customizable alternatives [1,2,3,4]. Additive manufacturing, especially fused filament fabrication (FFF), has emerged as a promising approach for producing patient-specific bone grafts with complex architectural features [5,6]. Fused filament fabrication employs a continuous filament during 3D printing, reducing material waste while keeping costs low and enabling the creation of complex designs, as well as compatibility with various materials [7]. Although FFF has been widely used for polymer-ceramic composites [8,9,10,11,12], its application for pure ceramic scaffolds introduces challenges related to filament development, printing parameters, and postprocessing techniques to obtain a hierarchical porosity [13,14,15]. Hydroxyapatite (HA), is the most widely used ceramic biomaterial in bone regeneration, as it resembles the mineral phase of vertebrate bone [16], and has a well-established biocompatibility and osteoconductivity [8,17]. However, HA possesses poor mechanical properties due to a low tensile and compressive strength, and difficulty in shaping, deeming this material unsuitable for tissue engineering purposes when used alone [18,19]. Thus, while bone tissue-engineered scaffolds have been extensively studied, the fabrication of pure HA scaffolds using FFF methods remains relatively underexplored [20].

The performance of bone substitutes depends on architectural features across multiple scales [21,22,23]: macro-architecture (scaffold shape and size); micro-architecture (internal porosity, pore interconnectivity, and infill parameters); and nano-architecture (surface topography and chemistry, often modified through post-processing such as sintering) [24]. All three levels influence the overall mechanical properties and osteoconduction capabilities of the 3D-printed scaffold [25,26]. Sintering plays a critical role in converting 3D-printed constructs into pure ceramic scaffolds. This process removes polymeric binders through a controlled thermal treatment [26,27,28], enhancing the mechanical strength and structural integrity of the scaffolds, while also increasing material bioactivity [29]. Preclinical studies have demonstrated the efficacy of sintered HA scaffolds in supporting osteoconduction and bone regeneration [25,30,31,32].

The novelty of this work lies in the use of a colloidal processing technique and sintering post-processing to produce highly loaded ceramic feedstock tailored for FFF. While the technical feasibility and physical properties of scaffolds manufactured via this method have been previously reported by Ferrández-Montero et al., 2024 [12], a comprehensive biological evaluation using advanced, standardized in vitro techniques has been lacking. This study addresses this critical gap by providing a detailed assessment of the osteogenic response in human-derived cells through multiplex immunoassays and reverse transcription–quantitative polymerase chain reaction (RT-qPCR)) Such analysis is essential to validate the bioactivity of these scaffolds and represents a critical step toward future in vivo evaluations and the eventual clinical translation of 3D-printed pure ceramic constructs.

Therefore, the primary objective of this study was to investigate the biological properties of MG-63 cells seeded onto sintered HA scaffolds, compared to MG-63 cells growing directly on the surface of culture plates over a 7-day incubation period. The specific objectives were to evaluate: (1) morphological changes through scanning electron microscopy (SEM); (2) cell viability using confocal scanning laser microscopy (CSLM); (3) cell proliferation assessed through tetrazolium assay via spectrophotometry; (4) cytotoxicity using a tetrazolium assay; (5) gene expression through reverse-transcription–quantitative polymerase chain reaction; (6) protein synthesis by multiplex immunoassay.

## 2. Materials and Methods

### 2.1. Study Design

This research involves a series of six in vitro assays to determine the structural and biological properties of sintered HA scaffolds (HAsint). MG-63 cells seeded onto the scaffold surfaces (test group) were used to assess biocompatibility and compared to MG-63 cells growing on the treated surface of the culture plates (negative control). Ethics approval was not required for this study since it did not involve human or animal subjects.

Each assay was performed with a total of three to six biological replicates for each study interval (24 h, 48 h, 72 h, and 7 days). Before each assay, scaffolds underwent sterilization via autoclaving (121 °C, 1.10 bar, 20 min) and were subsequently immersed in sterile phosphate-buffered saline (PBS) for 1 h. All scaffolds were seeded simultaneously, and after each experimental time point, the scaffolds were retrieved, assessed, and discarded.

### 2.2. Cell Culture

An experimental cell line of MG-63 cells (ATCC, CRL-1427) derived from human osteosarcoma was used for all the assays. The culture media consisted of Eagle’s Minimum Essential Medium with Earle’s Balanced Salt Solution (Sigma-Aldrich, Saint Louis, MO, USA), with 10% fetal bovine serum (Biowest, Nuaillé, France) and 1% penicillin–streptomycin (Gibco, Grand Island, NY, USA). The initial cell line was thawed according to the manufacturer’s protocol and seeded onto 75 cm^2^ flasks, which were then incubated at 37 °C in a humidified atmosphere containing 5% CO_2_.

After reaching a cell confluence of 80% or more, subcultures were obtained, and cell counts were performed for each assay. Cryopreservation was conducted using liquid nitrogen (−196 °C) and dimethyl sulfoxide (DMSO) as the cryoprotectant.

### 2.3. Scaffold Design and Fabrication

Commercial HA-containing filaments (FOss HA, COLFEED4Print S.L., Madrid, Spain) with a diameter of 1.75 mm and a nominal HA particle content of 50 vol.% were used to fabricate custom 3D scaffolds. The filaments consist of homogeneously dispersed HA phase within a polylactic acid (PLA) thermoplastic matrix, produced via a colloidal processing method [12,33]. The technical characterization of the colloidal feedstock used in the present research has been previously reported by Ferrández-Montero et al., 2024 [12].

**Figure 1:**
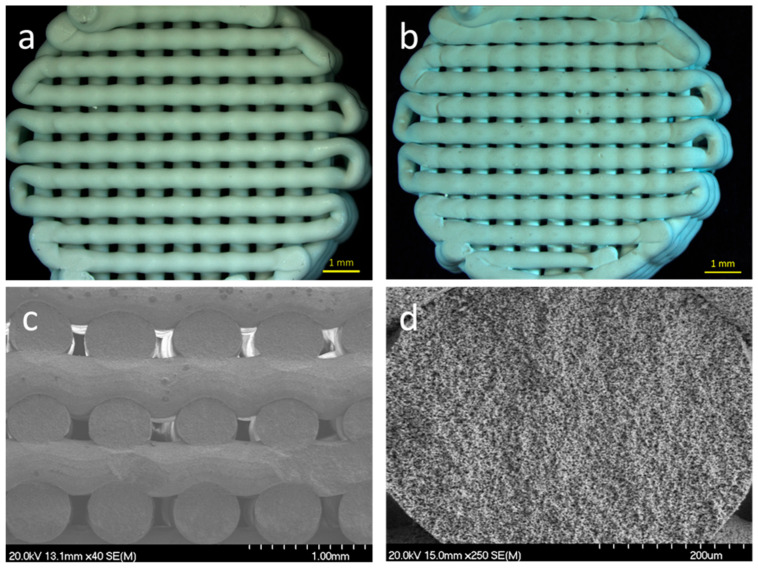
Scaffold design, with cylindrical geometry (9 mm diameter × 2.5 mm height) before sintering (**a**) and after sintering (**b**). Structure of the sintered scaffold cross-section at ×40 magnification (**c**) and ×250 magnification (**d**).

A standardized 3D sintered scaffold morphology was employed for all experimental scaffolds, labeled as HAsint (Figure 1). Scaffolds were manufactured in a cylindrical shape measuring 9 mm in diameter and 2.5 mm in height. The FFF printer used was a NX PRO Dual Direct drive HR + PELLET printer (Tumaker, Vizcaya, Spain) with a nozzle diameter of 0.4 mm. Scaffold geometries were designed using 3D freeware (Tinkercad, Autodesk, San Francisco, CA, USA), and slicing was achieved with Ultimaker Cura v5.3.1 software (Ultimaker, Utrecht, The Netherlands), selecting linear infill patterns. Prior to printing, the bed and nozzle were set to 40 °C and 165 °C, respectively, with a filament feed rate of 20 mm/s.

The fabricated scaffolds were subsequently subjected to a thermal post-processing treatment consisting of a debinding and sintering cycle under an air atmosphere, in order to obtain a pure ceramic scaffold [34,35]. This treatment removes the PLA polymer component and creates an open porous ceramic structure. The thermal cycle involved heating the scaffolds at 1 °C/min up to 600 °C with a dwell time of 30 min; followed by an increase in the heating rate of 5 °C/min up to 1250 °C, with a holding time of 2 h. The porosity of the HAsint scaffolds was determined using the Archimedes method in water, obtaining the dry, submerged, and saturated masses. From these values, the relative density and total porosity of the material were calculated.

### 2.4. Experimental Assays

#### 2.4.1. Morphological Characteristics (Scanning Electron Microscopy—SEM)

The scaffold surfaces were morphologically analyzed using SEM. Cells were seeded onto each scaffold at an initial density of 2 × 10^4^ cells/well in 48-well plates and cultured in a humidified atmosphere (5% CO_2_, 37 °C) for 24, 72 h, and 7 days. Three biological replicates were analyzed.

After incubation, the medium was removed, and the scaffolds were fixed by immersion in a solution of 2.5% glutaraldehyde and 4% paraformaldehyde in PBS (pH 7.4) for 2 h at room temperature. After removal of the fixative solution, the scaffolds were stored in PBS overnight. Subsequently, sample dehydration was achieved through a graded series of distilled water solutions (30% to 95% dH_2_O). After dehydration, the specimens were dried using a critical point dryer (Leica EM CPD300, Leica Microsystems, Wetzlar, Germany) according to the manufacturer’s protocol. The dried samples were then sputter-coated with gold and examined under SEM (Hitachi S-4800 FEG-SEM, Tokyo, Japan) at an accelerating voltage of 7 kV to assess scaffold topography.

#### 2.4.2. Cell Viability (Confocal Scanning Laser Microscopy—CSLM)

Cell viability was evaluated using CLSM (Leica Microsystems, Wetzlar, Germany) with the LIVE/DEAD^®^ viability/Cytotoxicity kit (Invitrogen, Thermo Fisher Scientific, Waltham, MA, USA). For each scaffold type and time point (24 h and 7 days), three biological replicates were analyzed.

Cells were seeded on scaffolds at 2 × 10^4^ cells/well in 48-well plates and kept under standard culture conditions (5% CO_2_, 37 °C). Following incubation, scaffolds were rinsed with culture media to minimize background esterase activity. A working solution was prepared by combining 10 mL of PBS, 20 µL of 2 mM EthD-1 (with a final concentration of 4 µM), and 5 µL of calcein AM (yielding a final concentration of 2 µM). After thorough mixing, 150 µL of the solution was added to each well and incubated at room temperature for 30 min.

Confocal laser scanning microscopy was performed using a FITC filter (Optolong Optics Co., Ltd., Kunming, China) for calcein AM (Excitation/Emission 494/517 nm) and a RFP filter (Lifelt Filter Co., Ltd., Xinxiang, China) for EthD-1 (Excitation/Emission 528/617). A negative control, consisting of methanol-fixed (70% for 15 min) dead cell scaffolds, was included to validate staining specificity and solution performance.

#### 2.4.3. Cell Proliferation (Tetrazolium Assay WST-1)

Cell proliferation was evaluated using a water-soluble tetrazolium salt (WST-1) assay (Cell Proliferation Reagent, Roche, Basel, Switzerland) at 24, 48, 72 h, and 7 days post-seeding. Six biological replicates were analyzed for each scaffold type and time point.

Cells were seeded at 2 × 10^4^ cells/well onto scaffolds and maintained under standard culture conditions. At each time point, scaffolds were transferred to fresh wells containing 800 µL culture medium, and then 80 µL WST-1 reagent was added to each well and incubated for 4 h. Plates were gently agitated for 1 min, and 100 µL supernatant was transferred to a new plate. Absorbance was measured at 440 nm (test wavelength, A_440_) and 650 nm (reference wavelength, A_650_) using a microplate reader (Multiskan SkyHigh, Thermo Scientific, Waltham, MA, USA). Cell proliferation was calculated as: (A_440_ − A_650_) − A_blank_, where A_blank_ represents the absorbance from scaffold-containing wells without cells. Results are presented as mean values of all biological replicates.

#### 2.4.4. Cytotoxicity (Tetrazolium Assay WST-1)

Cytotoxicity was assessed both with a direct and an indirect method. The direct method consisted of culturing cells directly on the scaffold surface, while the indirect method involved culturing cells with degradation media obtained by immersing the scaffolds in culture medium for predetermined time intervals. The indirect cytotoxicity assessment aimed to evaluate any potential leachable byproducts that might affect cell viability. Cytotoxicity was assessed using the WST-1 assay (Cell Proliferation Reagent, Roche, Basel, Switzerland), following the established protocol described in the cell proliferation assay section. Both methods included six biological replicates.

For the direct assessment, MG-63 cells were seeded onto scaffolds at an initial density of 1 × 10^5^ cells/well and cultured for 24 h, 48 h, 72 h, and 7 days.

For the indirect assessment, scaffolds were first incubated in culture medium (without cells) for the same time intervals to obtain degradation products. Subsequently, 2 × 10^4^ MG-63 cells/well were seeded into this conditioned medium for 24 h. After that, the medium was discarded, and the cells were rinsed with PBS (the control group used media obtained after the same time intervals but without scaffold submersion). The indirect cytotoxicity was measured by obtaining 100 µL of supernatant (1:2 dilution), and a spectrophotometric assessment was carried out.

In both cytotoxicity assays, absorbance was measured with a microplate reader (Multiskan SkiHigh, Thermo Scientific, Waltham, MA, USA) and cytotoxicity was calculated as (A_440_ − A_650_) − A_blank_ and presented as mean values of all biological replicates.

#### 2.4.5. Gene Expression (Reverse-Transcription–Quantitative Polymerase Chain Reaction)

Osteogenic gene expression was analyzed using reverse transcription–quantitative polymerase chain reaction (RT-qPCR) on the LightCycler 480 (Roche, Basel, Switzerland). MG-63 cells (3 × 10^4^ cells/well) were cultured on scaffolds for 24 h, 48 h, 72 h, and 7 days, with cells grown directly on well plates serving as controls.

Total RNA was extracted using the RNeasy Mini Kit (Qiagen, Hilden, Germany) and quantified by spectrophotometry (NanoDrop, Thermo Scientific, Waltham, MA, USA). Each biological replicate consisted of pooled RNA from 5 to 8 scaffolds. Reverse transcription was performed on 800 ng RNA using SuperScript^TM^ IV VILO^TM^ Master Mix with ezDNase (Invitrogen, Thermo Fisher Scientific, Waltham, MA, USA) treatment. The RT-qPCR analysis employed KAPA SYBR^®^ FAST reagents (KAPA BioSystems, Wilmington, MA, USA), with gene expression normalized to TATA-box binding protein (TBP) and Glyceraldehyde 3-phosphate dehydrogenase (GAPDH) reference genes and calculated via the 2^−ΔΔ*Ct*^ method. Primer sequences are detailed in Table A1.

#### 2.4.6. Protein Synthesis (Multiplex Immunoassay)

Secreted proteins matrix metalloproteinase 1 (MMP-1), receptor activator of nuclear factor kappa-B ligand (RANKL), macrophage colony-stimulating factor (M-CSF), interleukin (IL)-6, and IL-8 were measured using Luminex^®^ multiplex assays (Luminex200, Luminex Corporation, Austin, TX, USA). Protein analysis was performed using specific panels: MILLIPLEX^®^ Human Cytokine/Chemokine/Growth Factor Panel A (HCYTA-60K-03) (Merck KGaA, Darmstadt, Germany) for IL-6, IL-8, and M-CSF; MILLIPLEX^®^ MAP Human MMP Magnetic Bead Panel 2 (HMMP2MAG-55K-01) (Merck KGaA, Darmstadt, Germany) for MMP-1; and MILLIPLEX^®^ MAP Human RANKL Magnetic Bead—Single Plex (HRNKLMAG-51K-01) (Merck KGaA, Darmstadt, Germany) for RANKL. MG-63 cells (2 × 10^4^ cells/well) were seeded in 48-well plates and incubated (24 h, 48 h, 72 h, and 7 days). Subsequently, 900 µL of supernatant was collected at each interval from each of the four biological replicates and stored at −80 °C.

Samples were centrifuged to remove debris and analyzed undiluted per manufacturer protocols, except for MMP-1 assessment, where 1:4 dilutions were obtained for each sample prior to analysis. Data were processed using xPonent^®^ software (version 4.2, Luminex Corporation, Austin, TX, USA).

### 2.5. Data Analysis

#### 2.5.1. Outcome Variables

Results obtained from SEM and CLSM imaging were presented qualitatively through descriptive analysis, only for the test group. Quantitative data for the cell proliferation and cytotoxicity assays were measured in absorbance units (AU), while relative gene expression levels were quantified as relative fold changes using the 2^−ΔΔ*Ct*^ method. Protein synthesis was determined at concentrations of pg/mL.

#### 2.5.2. Statistical Analysis

Statistical analyses were performed to evaluate the experimental data. First, normality was assessed using the Shapiro–Wilk test, supplemented by evaluation of distribution parameters (skewness and kurtosis) and visual inspection of boxplot diagrams. All data are presented as mean and standard deviation (SD).

For comparative analyses, one-way ANOVA was used to examine differences between scaffold and control groups across the different incubation periods for the results obtained in the cell proliferation, cytotoxicity, relative gene expression, and protein synthesis assays. Based on the variance homogeneity results, post hoc comparisons were conducted using either Bonferroni’s test or Dunnett’s multiple comparisons test (based on homogeneity of variances). A significance threshold of *p* < 0.05 was applied for all statistical tests. All analyses were performed using IBM SPSS Statistics software (version 29.0.1.1, IBM Corporation, Armonk, NY, USA).

## 3. Results

### 3.1. Morphological Characteristics (Scanning Electron Microscopy—SEM)

**Figure 2:**
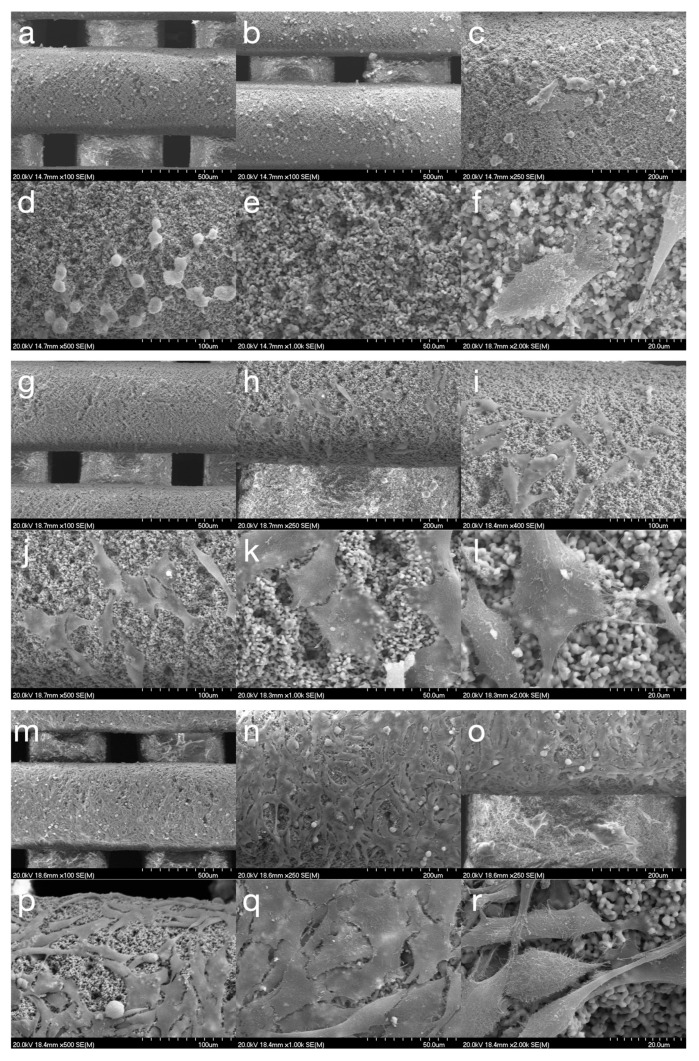
Scanning electron microscopy analysis of MG-63 cells co-cultured with HAsint scaffolds. Representative micrographs captured at (**a**–**f**) 24 h, (**g**–**l**) 72 h, and (**m**–**r**) 7 days post-seeding, shown at magnifications ranging from ×50 to ×2000. MG-63 cells demonstrated a progressive morphological adaptation to the scaffold surface, with a distinct transition to spindle-shaped morphology first observed at 72 h post-seeding.

During the culture period, MG-63 cells showed clear changes in shape and how they were arranged. Within the first 24 h after seeding, cells mostly appeared rounded with few extensions, typical of the initial attachment stage. By 72 h, they shifted to a spindle shape. This change in shape was linked to the formation of cytoplasmic processes that created interconnected networks between neighboring cells and anchored into the scaffold’s porous structure. After one week of incubation, SEM analysis revealed increased cell coverage (more than 90% of the scaffold surface), ongoing morphological development with flattened, polygonal cells, and the formation of a dense layer with overlapping processes (Figure 2).

Struts of the sintered scaffolds exhibited a relative density of 56% (as measured using the Archimedes method in water). The corresponding total porosity of 44% was estimated to be 40% of open porosity and 4% of closed one.

### 3.2. Cell Viability (Confocal Scanning Laser Microscopy—CSLM)

**Figure 3:**
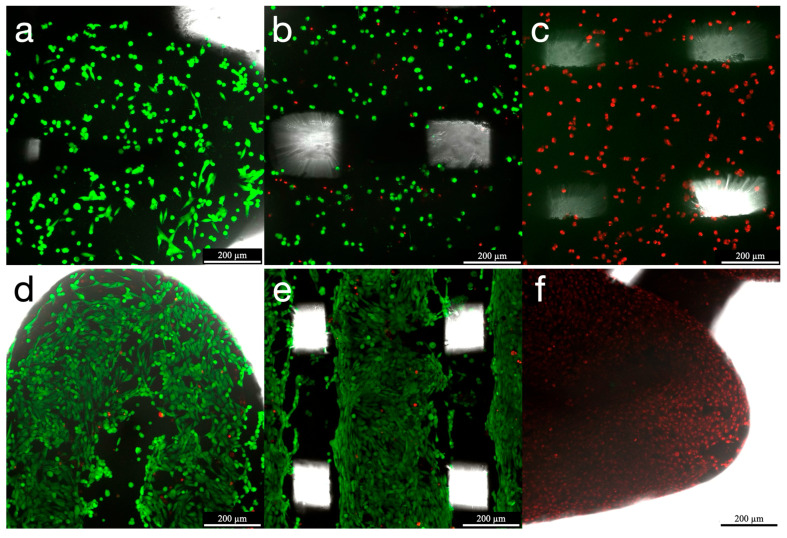
Confocal scanning microscopy imaging of MG-63 cells after LIVE/DEAD^®^ staining, seeded onto HAsint scaffolds after 24 h and 7 days of incubation. (**a**,**b**) HAsint scaffolds 24 h after seeding; (**c**) Negative control HAsint scaffolds 24 h after seeding. (**d**,**e**) HAsint scaffolds 7 days after seeding; (**f**) Negative control HAsint scaffolds 7 days after seeding. Live cells are shown in green and dead cells in red, with a magnification of ×10 (Scale bar, 200 µm).

LIVE/DEAD^®^ staining at 24 h revealed homogeneous cell distribution across scaffold surfaces, with a low proportion of dead cells. Negative controls demonstrated complete cell death, confirming the proper functionality of the staining and the validity of the assay. Viable cells exhibited characteristic spindle-shaped morphology with uniform cell attachment. After 7 days of incubation, an increase in cellular confluency was observed, along with maintained high cell viability. Some localized cell clusters can be observed within some specimens (Figure 3).

### 3.3. Cell Proliferation (Tetrazolium Assay WST-1)

The cell proliferation of MG-63 cells growing on both HAsint scaffolds and in 2D cultures showed statistically significant differences when comparing incubation times of 24 h and 7 days (*p* < 0.05). The comparison between groups indicates a significantly higher absorbance for the control group at all experimental time points. This difference was observed from the first assessment at 24 h with an absorbance of 0.42 AU (SD = 0.05) for the HAsint group and 1.32 AU (SD = 0.04) for the control group. At 1 week, the mean absorbance was 2.87 AU (SD = 0.23) for the test group and 4.78 AU (SD = 0.15) for the control group (Table 1).

### 3.4. Cytotoxicity (Tetrazolium Assay WST-1)

The indirect cytotoxicity assay showed no statistically significant differences in MG-63 cell viability after 24 h incubation in degradation media collected at the initial and final incubation intervals, neither in the scaffold group nor in the culture plate-adherent cells. Control cells grown directly on the plates maintained higher absorbance values at all measured intervals compared to cells growing on scaffolds (*p* < 0.05) (Table 2).

The direct cytotoxicity assay showed significant differences in viability throughout the 7-day incubation period. The HAsint group reached cell confluency over the scaffold surface after 48 h, with absorbance reaching 3.62 AU (SD = 0.21). This was followed by a significant reduction in cell viability after 7 days of incubation, to 1.72 AU (SD = 0.27) (Table 3).

### 3.5. Gene Expression (Reverse-Transcription–Quantitative Polymerase Chain Reaction)

**Figure 4:**
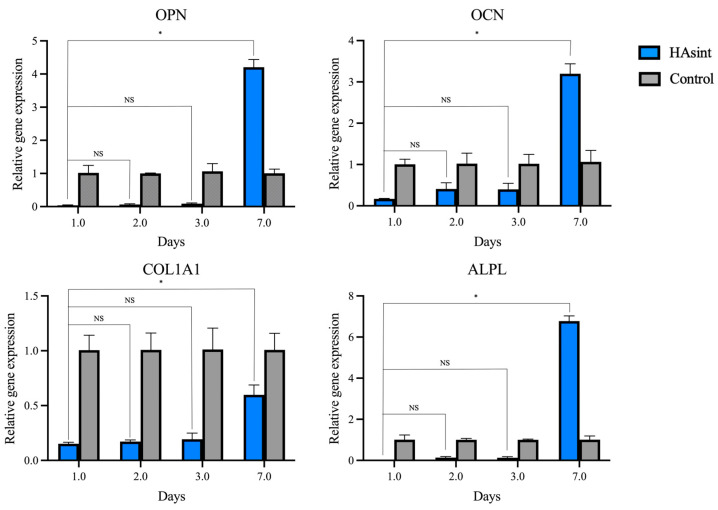
Relative gene expression profiles of osteogenic markers (OPN, OCN, COL1A1, ALPL) in MG-63 cells cultured on HAsint scaffolds, normalized to GADPH and TBP and analyzed by RT-qPCR over 7 days. Well-surface cultured cells served as controls. (OPN: osteopontin; OCN: osteocalcin; COL1A1: type 1 collagen; ALPL: alkaline phosphatase). Asterisks denote significant differences (* *p* < 0.05, NS no statistical significance).

Gene expression analysis confirmed that MG-63 cells seeded onto both HAsint, and the culture plate surfaces exhibited active osteogenic differentiation, as evidenced by significant mRNA expression of key markers: OCN, OPN, COL1A1, and ALPL (Figure 4).

The control group was used as a reference to determine the fold change in gene expression from the MG-63 cells proliferating over the HAsint scaffolds. The cells growing on scaffolds over HAsint showed expression levels of OPN, OCN, and ALPL that were 4.21, 3.20, and 6.77 times higher than those in the control group, respectively (*p* < 0.05). The expression level of ALPL was 0.60 times that of the control group (*p* < 0.05) (Table A2).

### 3.6. Protein Synthesis (Multiplex Immunoassay)

**Figure 5:**
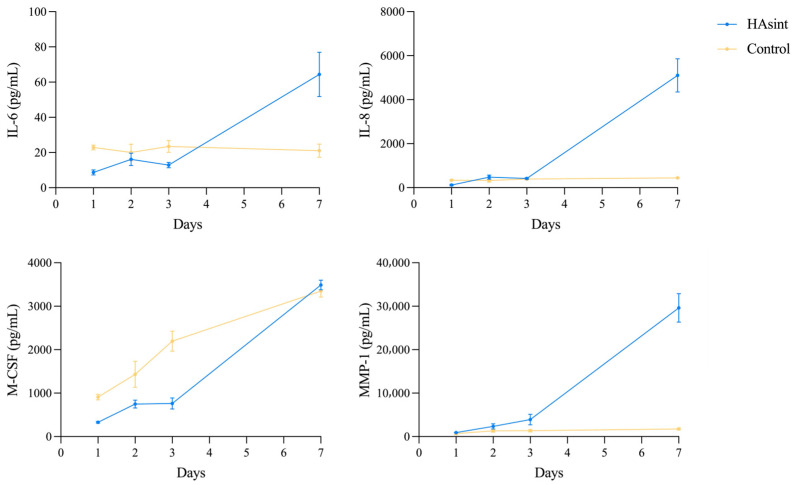
Protein synthesis profile of MG-63 cells. Concentrations (pg/mL) of IL-6, IL-8, M-CSF, and MMP-1 in culture supernatants were quantified at 24 h to 7 days using Luminex^®^ multiplex immunoassays. While RANKL was evaluated, its levels remained below the detection threshold at all time points. (IL-6: interleukin-6; IL-8: interleukin-8; M-CSF: macrophage colony-stimulating factor; MMP-1: matrix metalloproteinase-1).

Immunoassay analysis detected measurable concentrations of IL-6, IL-8, M-CSF, and MMP-1, while RANKL levels remained below detection limits throughout all incubation periods (Figure 5).

At the initial assessment (24 h), IL-6, IL-8, and M-CSF showed significantly lower protein concentrations (*p* < 0.05) in the HAsint group compared to the control (IL-6: 8.68 pg/mL [SD = 1.44] vs. 22.91 pg/mL [SD = 1.35]; IL-8: 115.82 pg/mL [SD = 31.02] vs. 338.10 pg/mL [SD = 20.06]; M-CSF: 327.62 pg/mL [SD = 20.14] vs. 908.80 pg/mL [SD = 63.56]). Only MMP-1 showed significantly higher concentrations in the HAsint group at 24 h (Table A3). At 48 h and 72 h, there were no statistically significant differences in analyte levels between the HAsint scaffolds and the control group (*p* > 0.05), except for M-CSF. 

Throughout the 7-day incubation period, all detected analytes in the HAsint group showed a significant increase in secreted concentrations compared to baseline measurements (*p* < 0.05) and were significantly higher than the controls for IL-6, IL-8, and MMP-1 at the final time point (IL-6: 64.35 pg/mL [SD = 12.57] vs. 21.05 pg/mL [SD = 3.77]; IL-8: 4970.39 pg/mL [SD = 756.44] vs. 444.07 pg/mL [SD = 9.67]; MMP-1: 29,626.07 pg/mL [SD = 3271.42] vs. 1745.65 pg/mL [SD = 254.07]). 

## 4. Discussion

An in vitro study was conducted to systematically assess the biological performance of sintered HA scaffolds for their potential use as bone tissue engineering constructs in regenerative applications. 

The fabrication of scaffolds for bone tissue engineering purposes is a complex process that requires optimization of micro- and nano-architecture to balance biological functionality with structural integrity of the construct [32]. While highly porous HA scaffolds with pore sizes ranging from 100 to 400 µm have been widely investigated for their ability to support bone regeneration [36,37], conventional fabrication techniques, such as binder jetting, robocasting, laser-assisted gelling, and material jetting, often suffer from limitations in dimensional accuracy, surface quality, and resolution [38].

Furthermore, the 3D printing of pure HA scaffolds, as opposed to polymer-ceramic composites, remains particularly challenging due to issues related to printability, such as poor particle bonding and inadequate flowability during extrusion [39,40,41]. In this study, these challenges were addressed through a colloidal FFF approach, which enable the production of a highly loaded HA feedstock that, with subsequent debinding and sintering, yielded a pure ceramic scaffold [35]. An additional advantage of such purely ceramic scaffolds is their compatibility with steam sterilization, a widely used, reliable, and residue-free method that ensures deep penetration and eliminates risks of toxic residues [42,43]. This represents a significant benefit over polymer-ceramic composites, which may degrade or deform under moist or heat sterilization [10].

The use of the MG-63 cell line was selected for this study as a well-established model in bone biomaterial research [44]. This line exhibits stable osteoblast-like characteristics, including the ability to mineralize and express key bone-related markers, making it a reproducible tool for initial biocompatibility testing [45]. However, we acknowledge that as an osteosarcoma-derived cell line, MG-63 cells may not fully replicate the physiological behavior of primary human osteoblasts or mesenchymal stem cells. This represents a limitation in the present study. Discrepancies in outcomes across bone tissue engineering research often stem from the use of different cellular models (ranging from murine MC3T3 to MG-63 and mesenchymal stem cells), and are combined with a lack of standardization in analytical protocols [46].

Cell attachment and spreading are crucial early-stage interactions between cells and scaffolds. In this study, the observation of a homogeneous distribution of spindle-shaped cells across the scaffold surface, along with their extensive intercellular connections, indicates favorable adaptation to the 3D environment. This morphological assessment reflects the typical behavior of MG-63 cells during osteogenic differentiation in native bone environments [46,47].

As expected, in the cell proliferation and cytotoxicity assays, the control group showed significantly higher absorbance compared to the HAsint scaffolds, since cells cultured on the treated plate surfaces benefit from ideal growth conditions. Despite this difference, both groups exhibited a consistent increase in proliferation over time, indicating sustained cell growth across all samples. These findings align with previous studies. Wu et al. obtained similar results using a WST-8 reagent, demonstrating continuous cell proliferation over sintered HA scaffolds for 5 days [32]. Liu et al. reported increased cell activity and osteogenicity on similar HA scaffolds obtained through digital light processing additive manufacturing, as compared to a blank group [48].

The cytotoxicity assessment revealed distinct responses between indirect and direct assays. While MG-63 cell viability remained unaffected in degradation media collected from scaffolds across increasing incubation times (indirect assay), a significant reduction was observed when cells were cultured directly in the scaffolds (direct assessment). This apparent discrepancy may be attributed to in vitro experimental conditions, particularly the high scaffold surface-area-to-media-volume ratio employed in this methodology. Under these conditions, HA solubility limits were likely exceeded, especially during prolonged incubation times, resulting in solution oversaturation and subsequent HA reprecipitation [49]. This ion release and reprecipitation could be associated with cytotoxicity in the local environment of the scaffolds in the direct assay, explaining the observed differences in cytotoxic effects. Alternatively, reports in the literature indicate that the cytotoxicity evaluation for ceramics of high specific surface area may lead to false negative results due to the intense adsorption of crucial ions from the culture medium, leading to changes in extracellular ion concentrations [50,51].

The current literature lacks a comprehensive analysis of how pure HA scaffolds influence osteogenic gene expression and protein secretion profiles, creating a notable gap in understanding their biological mechanisms. In this study, the observed upregulation of osteogenic gene expression (OCN, OPN, ALPL), along with an increase in protein secretion (IL-6, IL-8, MMP-1), suggests that 3D pure hydroxyapatite constructs enhance the osteogenic potential of MG-63 cells beyond conventional 2D cultures. These findings are consistent with previous reports on similar biomaterials [52,53], and mirror the natural progression of osteogenic differentiation, where early collagen synthesis is followed by increased non-collagenous protein production and alkaline phosphatase activity during matrix maturation [54]. The increase in IL-6 and IL-8 observed at day 7, however, can be interpreted from a dual perspective. These interleukins play roles in bone physiology, osteogenesis, angiogenesis, and osteoclastogenesis [55], but elevated levels of these cytokines could also indicate an early inflammatory response to the scaffolds and their degradation byproducts.

The use of pure ceramic HA scaffolds in clinical settings has been previously reported. Mangano et al. described the 7-year follow-up after alveolar bone reconstruction using a 3D printed ceramic scaffold obtained through rapid prototyping, dispense-plotting, and sintering. The analysis by histomorphometry and micro-computed tomography demonstrated complete integration of the ceramic, signs of biomaterial degradation and resorption by multinucleated giant cells, and a 23% volume decrease over time [56]. Nevertheless, the implementation of 3D printed materials for human use is contingent on regulatory approvals, which vary across regional jurisdictions [7].

A key limitation of this study is the absence of an optimal 3D control for the HAsint scaffolds, as plate-adherent cell cultures (2D) do not provide a physiologically relevant comparison to a three-dimensional growth environment. Ideally, a control group would consist of biologically inert polymeric or ceramic scaffolds with known neutral effects on MG-63 cells and that mimic the porosity and surface roughness of HA scaffolds. However, to the best of our knowledge, such a material is not currently feasible to obtain, as most reference materials exhibit some degree of bioactivity or biodegradability. While this constraint restricts direct intergroup comparisons, the intragroup temporal analysis across experimental time points consistently demonstrates robust biocompatibility and osteogenic potential. An additional limitation arose from the manufacturing complexities in the sintering process, which limited batch yields, consequently reducing the experimental sample size. Furthermore, additional technical characterization, including the quantitative determination of porosity and mechanical properties, would have provided valuable structure correlations to better elucidate the biological results. These constraints should be considered when extrapolating the findings to in vivo settings.

The demonstrated in vitro biocompatibility and bioactivity of HAsint scaffolds establish a foundation for translational applications, although critical evaluation of immune and inflammatory responses, along with long-term safety assessments in animal models, remains essential. Future studies should explore strategies such as functionalization with growth factors or stem cells, alongside in vivo evaluations, to bridge the gap toward clinical translation.

## 5. Conclusions

This study demonstrates the biocompatibility of 3D-printed HAsint scaffolds, evidenced by sustained cell proliferation and attachment, along with increased osteogenic gene expression and protein synthesis in MG-63 cells growing on the 3D-printed scaffolds compared to 2D cultures on culture plates. These features position HAsint scaffolds as promising candidates for bone tissue engineering.

## Figures and Tables

**Table 1:** Cell proliferation of HAsint scaffolds measured by spectrophotometry using a WST-1 assay, measured at 24, 48, 72 h, and 7 days. Cell proliferation was significantly increased in the control group; however, the test group showed consistent proliferation throughout the study period. One-way ANOVA test and Dunnett’s multiple comparisons test.

Cell Proliferation Assay								
Infill	N	Absorbance (Mean SD)				*p* Value (Between Timepoints)		
		24 h	48 h	72 h	7 D	24 h vs. 48 h	24 h vs. 72 h	24 h vs. 7 D
HAsint	6	0.42 (0.05)	0.88 (0.23)	1.40 (0.17)	2.87 (0.23)	0.049	<0.001	0.001
Control	3	1.32 (0.04)	2.47 (0.14)	3.30 (0.06)	4.78 (0.15)	0.017	<0.001	0.002
*p* value (between groups)		<0.001	<0.001	<0.001	<0.001			

**Table 2:** Indirect cytotoxicity assay using a WST-1 assay, measured through spectrophotometry on MG-63 cells incubated for 24 h on degradation media obtained after 24, 48, 72 h, and 7 days. One-way ANOVA and Bonferroni tests.

Indirect Cytotoxicity Assay								
Infill	N	Absorbance (Mean (SD))				*p* Value (Between Timepoints)		
		24 h	48 h	72 h	7 D	24 h vs. 48 h	24 h vs. 72 h	24 h vs. 7 D
HAsint	6	0.23 (0.02)	0.21 (0.01)	0.19 (0.01)	0.22 (0.02)	0.242	0.001	1.000
Control	3	0.28 (0.01)	0.27 (0.02)	0.27 (0.01)	0.32 (0.02)	1.000	1.000	0.235
*p* value (between groups)		0.002	<0.001	<0.001	<0.001			

**Table 3:** Direct cytotoxicity assessment of HAsint scaffolds. Evaluated using a WST-1 assay, absorbance was quantified through spectrophotometry at 24, 48, 72 h, and 7 days post-seeding. One-way ANOVA and Bonferroni tests.

Direct Cytotoxicity Assay								
Infill	N	Absorbance (Mean (SD))				*p* Value (Between Timepoints)		
		24 h	48 h	72 h	7 D	24 h vs. 48 h	24 h vs. 72 h	24 h vs. 7 D
HAsint	6	2.42 (0.22)	3.62 (0.21)	3.65 (0.26)	1.72 (0.27)	<0.001	<0.001	<0.001
Control	3	3.95 (0.17)	4.64 (0.18)	4.59 (0.22)	5.16 (0.07)	0.020	0.043	<0.001
*p* value (between groups)		<0.001	<0.001	<0.001	<0.001			

## Data Availability

The original contributions presented in the study are included in the article; further inquiries can be directed to the corresponding authors.

## Abbreviations

The following abbreviations are used in this manuscript:

CLSM	Confocal scanning laser microscopy
COL1A1	Collagen type 1
FFF	Fused filament fabrication
GAPDH	Glyceraldehyde 3-phosphate dehydrogenase
HA	Hydroxyapatite
HAsint	Sintered hydroxyapatite
IL	Interleukin
M-CSF	Macrophage colony-stimulating factor
MMP	Matrix metalloproteinase
PLA	Polylactic acid
RANKL	Receptor activator of nuclear factor kappa-B ligand
RT-qPCR	Reverse transcription–quantitative polymerase chain reaction
SEM	Scanning electron microscopy
TBP	TATA-box binding protein
WST	Water-soluble tetrazolium

## Appendix A

**Table A1:** Primer sequences used to amplify each target and housekeeping gene by RT-qPCR.

Gene	Primer	Sequence
TBP	Forward	5’-TGTATCCACAGTGAATCTTGGTTG-3’
	Reverse	5’-GGTTCGTGGCTCTCTTATCCTC-3’
GAPDH	Forward	5’-GTCTCCTCTGACTTCAACAGCG-3’
	Reverse	5’-ACCACCCTGTTGCTGTAGCCAA-3’
OPN	Forward	5’-CGAGGTGATATAGTGTGGTTTATGG-3’
	Reverse	5’-GCACCATTCAACTCCTCGCTTTC-3’
OCN	Forward	5’-CGCTACCTGTATCAATGGCTGG-3’
	Reverse	5’-CTCCTGAAAGCCGATGTGGTCA-3’
COL1A1	Forward	5’-GATTCCCTGGACCTAAAGGTGC-3’
	Reverse	5’-AGCCTCTCCATCTTTGCCAGCA-3’
ALPL	Forward	5’-GCTGTAAGGACATCGCCTACCA-3’
	Reverse	5’-CCTGGCTTTCTCGTCACTCTCA-3’

**Table A2:** Gene expression fold changes in cells cultured on HAsint scaffolds relative to control cells using the 2^−ΔΔ*Ct*^ method. One-way ANOVA and Bonferroni tests (for OCN and COL1A1) and Dunnett’s multiple comparisons test (for OPN and ALPL).

Gene Expression									
Gene	Infill	N	Relative Gene Expression (Mean (SD))				*p* Value (Between Timepoints)		
			24 h	48 h	72 h	7 D	24 h vs. 48 h	24 h vs. 72 h	24 h vs. 7 D
OPN	HAsint	3	0.04 (0.01)	0.07 (0.02)	0.09 (0.02)	4.21 (0.23)	0.764	0.337	0.005
	Control	3	1.02 (0.22)	1.00 (0.01)	1.06 (0.23)	1.00 (0.12)	1.000	1.000	1.000
	*p* value (between groups)		0.090	<0.001	0.096	0.002			
OCN	HAsint	3	0.17 (0.01)	0.41 (0.15)	0.40 (0.15)	3.20 (0.24)	1.000	1.000	<0.001
	Control	3	1.00 (0.12)	1.02 (0.26)	1.02 (0.23)	1.07 (0.27)	1.000	1.000	1.000
	*p* value (between groups)		0.002	0.043	0.039	<0.001			
COL1A1	HAsint	3	0.15 (0.01)	0.17 (0.02)	0.19 (0.06)	0.60 (0.09)	1.000	1.000	0.009
	Control	3	1.00 (0.14)	1.00 (0.15)	1.01 (0.20)	1.00 (0.15)	1.000	1.000	1.000
	*p* value (between groups)		<0.001	<0.001	<0.001	0.019			
ALPL	HAsint	3	0.00 (0.00)	0.13 (0.06)	0.13 (0.05)	6.77 (0.26)	0.283	0.283	0.049
	Control	3	1.01 (0.22)	1.00 (0.07)	1.00 (0.03)	1.01 (0.18)	1.000	1.000	1.000
	*p* value (between groups)		0.281	0.030	0.002	0.015			

**Table A3:** Protein secretion profiles of IL-6, IL-8, M-CSF, and MMP-1 by MG-63 cells cultured on HAsint scaffolds, quantified via Luminex^®^ immunoassay at 24 h, 48 h, 72 h, and 7 days. Statistical analysis: one-way ANOVA with Bonferroni post hoc test (M-CSF) and Dunnett’s multiple comparisons tests (IL-6, IL-8, and MMP-1).

Protein Synthesis									
Protein	Infill	N	Concentration pg/mL (Mean (SD))				*p* Value (Between Timepoints)		
			24 h	48 h	72 h	7 D	24 h vs. 48 h	24 h vs. 72 h	24 h vs. 7 D
IL-6	HAsint	4	8.68 (1.44)	16.16 (3.53)	12.87 (1.49)	64.35 (12.57)	0.165	0.092	0.024
	Control	3	22.91 (1.35)	20.03 (4.72)	23.43 (3.33)	21.05 (3.77)	0.985	1.000	0.998
	*p* value (between groups)		<0.001	0.973	0.135	0.038			
IL-8	HAsint	4	115.82 (31.02)	475.85 (95.66)	417.52 (32.70)	4970.39 (756.44)	0.029	<0.001	0.008
	Control	3	338.10 (20.06)	326.54 (77.52)	392.99 (37.52)	444.07 (9.67)	1.000	0.613	0.034
	*p* value (between groups)		0.001	0.570	0.998	0.010			
M-CSF	HAsint	4	327.62 (20.14)	748.23 (91.14)	762.60 (129.08)	3488.53 (109.91)	0.020	0.015	<0.001
	Control	3	908.80 (63.56)	1431.56 (299.87)	2194,41 (231.74)	3347.19 (137.61)	0.010	<0.001	<0.001
	*p* value (between groups)		0.002	<0.001	<0.001	1.000			
MMP-1	HAsint	4	910.23 (71.25)	2350.36 (593.97)	3921.86 (1209.48)	29,626.07 (3271.42)	0.259	0.249	0.023
	Control	3	679.47 (79.49)	1332.80 (319.91)	1341.54 (288.40)	1745.65 (254.07)	0.490	0.434	0.112
	*p* value (between groups)		0.950	0.482	0.316	0.024			

## References

[1] El-Rashidy A.A., Roether J.A., Harhaus L., Kneser U., Boccaccini A.R. (2017). Regenerating bone with bioactive glass scaffolds: A review of in vivo studies in bone defect models. Acta Biomater..

[2] Haugen H.J., Lyngstadaas S.P., Rossi F., Perale G. (2019). Bone grafts: Which is the ideal biomaterial?. J. Clin. Periodontol..

[3] Kolk A., Handschel J., Drescher W., Rothamel D., Kloss F., Blessmann M., Heiland M., Wolff K.-D., Smeets R. (2012). Current trends and future perspectives of bone substitute materials—From space holders to innovative biomaterials. J. Craniomaxillofac. Surg..

[4] Sanz M., Dahlin C., Apatzidou D., Artzi Z., Bozic D., Calciolari E., De Bruyn H., Dommisch H., Donos N., Eickholz P. (2019). Biomaterials and regenerative technologies used in bone regeneration in the craniomaxillofacial region: Consensus report of group 2 of the 15th European Workshop on Periodontology on Bone Regeneration. J. Clin. Periodontol..

[5] Gaharwar A.K., Singh I., Khademhosseini A. (2020). Engineered biomaterials for in situ tissue regeneration. Nat. Rev. Mater..

[6] Wang X., Mu M., Yan J., Han B., Ye R., Guo G. (2024). 3D printing materials and 3D printed surgical devices in oral and maxillofacial surgery: Design, workflow and effectiveness. Regen. Biomater..

[7] Ivanovski S., Breik O., Carluccio D., Alayan J., Staples R., Vaquette C. (2023). 3D printing for bone regeneration: Challenges and opportunities for achieving predictability. Periodontol. 2000.

[8] Soleymani S., Naghib S.M. (2023). 3D and 4D printing hydroxyapatite-based scaffolds for bone tissue engineering and regeneration. Heliyon.

[9] Zhang Y., Wang J., Ma Y., Han B., Niu X., Liu J., Gao L., Wang J., Zhai X., Chu K. (2018). Preparation of poly(lactic acid)/sintered hydroxyapatite composite biomaterial by supercritical CO_2_. Biomed. Mater. Eng..

[10] Shan E., Chamorro C., Ferrández-Montero A., Martin-Rodriguez R.M., Ferrari B., Sanchez-Herencia A.J., Virto L., Marín M.J., Figuero E., Sanz M. (2025). In Vitro Biological Properties Assessment of 3D-Printed Hydroxyapatite-Polylactic Acid Scaffolds Intended for Bone Regeneration. J. Funct. Biomater..

[11] Hutmacher D.W., Schantz J.T., Lam C.X., Tan K.C., Lim T.C. (2007). State of the art and future directions of scaffold-based bone engineering from a biomaterials perspective. J. Tissue Eng. Regen. Med..

[12] Ferrández-Montero A., Ortega-Columbrans P., Eguiluz A., Sanchez-Herencia A.J., Detsch R., Boccaccini A.R., Ferrari B. (2024). Biocompatible colloidal feedstock for material extrusion processing of bioceramic-based scaffolds. Polym. Compos..

[13] Mathiazhagan N., Palaniyappan S., Sivakumar N.K. (2023). Effect of fused filament fabrication parameters on crashworthiness studies of hydroxyapatite particle reinforced PLA composite thin-walled tubes. J. Mech. Behav. Biomed. Mater..

[14] Winarso R., Anggoro P.W., Ismail R., Jamari J., Bayuseno A.P. (2022). Application of fused deposition modeling (FDM) on bone scaffold manufacturing process: A review. Heliyon.

[15] Li Y.-C., Zhang Y.S., Akpek A., Shin S.R., Khademhosseini A. (2017). 4D bioprinting: The next-generation technology for biofabrication enabled by stimuli-responsive materials. Biofabrication.

[16] Dorozhkin S.V., Epple M. (2002). Biological and Medical Significance of Calcium Phosphates. Angew. Chem. Int. Ed..

[17] Tadic D., Epple M. (2004). A thorough physicochemical characterisation of 14 calcium phosphate-based bone substitution materials in comparison to natural bone. Biomaterials.

[18] Corcione C.E., Gervaso F., Scalera F., Montagna F., Maiullaro T., Sannino A., Maffezzoli A. (2017). 3D printing of hydroxyapatite polymer-based composites for bone tissue engineering. J. Polym. Eng..

[19] Ratnayake J.T.B., Mucalo M., Dias G.J. (2017). Substituted hydroxyapatites for bone regeneration: A review of current trends. J. Biomed. Mater. Res. B Appl. Biomater..

[20] Zhao R., Yang R., Cooper P.R., Khurshid Z., Shavandi A., Ratnayake J. (2021). Bone Grafts and Substitutes in Dentistry: A Review of Current Trends and Developments. Molecules.

[21] Carotenuto F., Politi S., Ul Haq A., De Matteis F., Tamburri E., Terranova M.L., Teodori L., Pasquo A., Di Nardo P. (2022). From Soft to Hard Biomimetic Materials: Tuning Micro/Nano-Architecture of Scaffolds for Tissue Regeneration. Micromachines.

[22] Cordell J.M., Vogl M.L., Wagoner Johnson A.J. (2009). The influence of micropore size on the mechanical properties of bulk hydroxyapatite and hydroxyapatite scaffolds. J. Mech. Behav. Biomed. Mater..

[23] Wang S., Kowal T.J., Marei M.K., Falk M.M., Jain H. (2013). Nanoporosity significantly enhances the biological performance of engineered glass tissue scaffolds. Tissue Eng. Part A.

[24] Chia H.N., Wu B.M. (2015). Recent advances in 3D printing of biomaterials. J. Biol. Eng..

[25] Ghayor C., Bhattacharya I., Guerrero J., Ozcan M., Weber F.E. (2022). 3D-Printed HA-Based Scaffolds for Bone Regeneration: Microporosity, Osteoconduction and Osteoclastic Resorption. Materials.

[26] Vivanco J., Slane J., Nay R., Simpson A., Ploeg H.L. (2011). The effect of sintering temperature on the microstructure and mechanical properties of a bioceramic bone scaffold. J. Mech. Behav. Biomed. Mater..

[27] Pramanik S., Agarwal A.K., Rai K.N., Garg A. (2007). Development of high strength hydroxyapatite by solid-state-sintering process. Ceram. Int..

[28] Pei X., Ma L., Zhang B., Sun J., Sun Y., Fan Y., Gou Z., Zhou C., Zhang X. (2017). Creating hierarchical porosity hydroxyapatite scaffolds with osteoinduction by three-dimensional printing and microwave sintering. Biofabrication.

[29] Bertone P.M., Olevsky L.M., Kathir K., Agnew S.A., Scheideler W.J., Hixon K.R. (2025). Sintering 3D-Printed Hydroxyapatite-Wollastonite Lattices Improve Bioactivity and Mechanical Integrity for Bone Composite Scaffolds. bioRxiv.

[30] Kim C., Lee J.W., Heo J.H., Park C., Kim D.H., Yi G.S., Kang H.C., Jung H.S., Shin H., Lee J.H. (2022). Natural bone-mimicking nanopore-incorporated hydroxyapatite scaffolds for enhanced bone tissue regeneration. Biomater. Res..

[31] Patel P.P., Buckley C., Taylor B.L., Sahyoun C.C., Patel S.D., Mont A.J., Mai L., Patel S., Freeman J.W. (2019). Mechanical and biological evaluation of a hydroxyapatite-reinforced scaffold for bone regeneration. J. Biomed. Mater. Res. Part A.

[32] Wu Q., Zhang X., Wu B., Huang W. (2013). Effects of microwave sintering on the properties of porous hydroxyapatite scaffolds. Ceram. Int..

[33] Ferrandez-Montero A., Lieblich M., Benavente R., González-Carrasco J.L., Ferrari B. (2020). New approach to improve polymer-Mg interface in biodegradable PLA/Mg composites through particle surface modification. Surf. Coat. Technol..

[34] Esposito Corcione C., Gervaso F., Scalera F., Padmanabhan S.K., Madaghiele M., Montagna F., Sannino A., Licciulli A., Maffezzoli A. (2019). Highly loaded hydroxyapatite microsphere/PLA porous scaffolds obtained by fused deposition modelling. Ceram. Int..

[35] Chirico C., Ferrández-Montero A., Eguiluz Á., Ortega-Columbrans P., Sanchez-Herencia A.J., Ferrari B. (2025). Colloidal approach to fabricate high-loaded feedstocks for material extrusion of dense sintered Al_2_O_3_ structures for biomedical applications. Bol. Soc. Espa. Cerám. Vidr..

[36] Schliephake H., Neukam F.W., Klosa D. (1991). Influence of pore dimensions on bone ingrowth into porous hydroxylapatite blocks used as bone graft substitutes: A histometric study. Int. J. Oral Maxillofac. Surg..

[37] Wilson C.E., de Bruijn J.D., van Blitterswijk C.A., Verbout A.J., Dhert W.J. (2004). Design and fabrication of standardized hydroxyapatite scaffolds with a defined macro-architecture by rapid prototyping for bone-tissue-engineering research. J. Biomed. Mater. Res. Part A.

[38] Marques A., Miranda G., Silva F., Pinto P., Carvalho Ó. (2021). Review on current limits and potentialities of technologies for biomedical ceramic scaffolds production. J. Biomed. Mater. Res. Part B Appl. Biomater..

[39] Bogala M.R. (2022). Three-dimensional (3D) printing of hydroxyapatite-based scaffolds: A review. Bioprinting.

[40] Kumar A., Kargozar S., Baino F., Han S.S. (2019). Additive Manufacturing Methods for Producing Hydroxyapatite and Hydroxyapatite-Based Composite Scaffolds: A Review. Front. Mater..

[41] Butscher A., Bohner M., Roth C., Ernstberger A., Heuberger R., Doebelin N., von Rohr P.R., Müller R. (2012). Printability of calcium phosphate powders for three-dimensional printing of tissue engineering scaffolds. Acta Biomater..

[42] Gogolewski S., Mainil-Varlet P. (1996). The effect of thermal treatment on sterility, molecular and mechanical properties of various polylactides: I. Poly(l-lactide). Biomaterials.

[43] Dai Z., Ronholm J., Tian Y., Sethi B., Cao X. (2016). Sterilization techniques for biodegradable scaffolds in tissue engineering applications. J. Tissue Eng..

[44] Staehlke S., Rebl H., Nebe B. (2019). Phenotypic stability of the human MG-63 osteoblastic cell line at different passages. Cell Biol. Int..

[45] Verma S., Kumar N. (2010). Effect of biomimetic 3D environment of an injectable polymeric scaffold on MG-63 osteoblastic-cell response. Mater. Sci. Eng. C.

[46] Chatree K., Sriboonaied P., Phetkong C., Wattananit W., Chanchao C., Charoenpanich A. (2023). Distinctions in bone matrix nanostructure, composition, and formation between osteoblast-like cells, MG-63, and human mesenchymal stem cells, UE7T-13. Heliyon.

[47] Docheva D., Padula D., Popov C., Mutschler W., Clausen-Schaumann H., Schieker M. (2008). Researching into the cellular shape, volume and elasticity of mesenchymal stem cells, osteoblasts and osteosarcoma cells by atomic force microscopy. J. Cell Mol. Med..

[48] Liu Z., Liang H., Shi T., Xie D., Chen R., Han X., Shen L., Wang C., Tian Z. (2019). Additive manufacturing of hydroxyapatite bone scaffolds via digital light processing and in vitro compatibility. Ceram. Int..

[49] Retegi-Carrión S., Ferrandez-Montero A., Eguiluz A., Ferrari B., Abarrategi A. (2022). The Effect of Ca^2+^ and Mg^2+^ Ions Loaded at Degradable PLA Membranes on the Proliferation and Osteoinduction of MSCs. Polymers.

[50] Liu Y.K., Lu Q.Z., Pei R., Ji H.J., Zhou G.S., Zhao X.L., Tang R.K., Zhang M. (2009). The effect of extracellular calcium and inorganic phosphate on the growth and osteogenic differentiation of mesenchymal stem cells in vitro: Implication for bone tissue engineering. Biomed. Mater..

[51] Klimek K., Belcarz A., Pazik R., Sobierajska P., Han T., Wiglusz R.J., Ginalska G. (2016). “False” cytotoxicity of ions-adsorbing hydroxyapatite—Corrected method of cytotoxicity evaluation for ceramics of high specific surface area. Mater. Sci. Eng. C.

[52] Yoshida T., Kikuchi M., Koyama Y., Takakuda K. (2010). Osteogenic activity of MG63 cells on bone-like hydroxyapatite/collagen nanocomposite sponges. J. Mater. Sci. Mater. Med..

[53] Gregor A., Filova E., Novak M., Kronek J., Chlup H., Buzgo M., Blahnová V., Lukášová V., Bartoš M., Nečas A. (2017). Designing of PLA scaffolds for bone tissue replacement fabricated by ordinary commercial 3D printer. J. Biol. Eng..

[54] Born A.K., Rottmar M., Lischer S., Pleskova M., Bruinink A., Maniura-Weber K. (2009). Correlating cell architecture with osteogenesis: First steps towards live single cell monitoring. Eur. Cell Mater..

[55] Xu J., Yu L., Liu F., Wan L., Deng Z. (2023). The effect of cytokines on osteoblasts and osteoclasts in bone remodeling in osteoporosis: A review. Front. Immunol..

[56] Mangano C., Giuliani A., De Tullio I., Raspanti M., Piattelli A., Iezzi G. (2021). Case Report: Histological and Histomorphometrical Results of a 3-D Printed Biphasic Calcium Phosphate Ceramic 7 Years After Insertion in a Human Maxillary Alveolar Ridge. Front. Bioeng. Biotechnol..

